# TPFusion: Texture Preserving Fusion of Infrared and Visible Images via Dense Networks

**DOI:** 10.3390/e24020294

**Published:** 2022-02-19

**Authors:** Zhiguang Yang, Shan Zeng

**Affiliations:** School of Mathematics and Computer Science, Wuhan Polytechnic University, Wuhan 430023, China; zengshan1981@whpu.edu.cn

**Keywords:** infrared and visible image fusion, texture preserving, densely connected network

## Abstract

In this paper, we design an infrared (IR) and visible (VIS) image fusion via unsupervised dense networks, termed as TPFusion. Activity level measurements and fusion rules are indispensable parts of conventional image fusion methods. However, designing an appropriate fusion process is time-consuming and complicated. In recent years, deep learning-based methods are proposed to handle this problem. However, for multi-modality image fusion, using the same network cannot extract effective feature maps from source images that are obtained by different image sensors. In TPFusion, we can avoid this issue. At first, we extract the textural information of the source images. Then two densely connected networks are trained to fuse textural information and source image, respectively. By this way, we can preserve more textural details in the fused image. Moreover, loss functions we designed to constrain two densely connected convolutional networks are according to the characteristics of textural information and source images. Through our method, the fused image will obtain more textural information of source images. For proving the validity of our method, we implement comparison and ablation experiments from the qualitative and quantitative assessments. The ablation experiments prove the effectiveness of TPFusion. Being compared to existing advanced IR and VIS image fusion methods, our fusion results possess better fusion results in both objective and subjective aspects. To be specific, in qualitative comparisons, our fusion results have better contrast ratio and abundant textural details. In quantitative comparisons, TPFusion outperforms existing representative fusion methods.

## 1. Introduction

Multi-sensor image fusion is an effective technique fusing multi-source images into one image that contains complementary information for better visual understanding. In the last two decades, this technique has been widely applied in the field of non-destructive testing, biosensor, military monitoring, clinical diagnose, remote sensing, etc. [1].

Infrared and visible image fusion technology is one essential branch of multi-modality image fusion field. It has aroused much concern among the researchers. Infrared image (IR) which gets from infrared sensor presents thermal radiation information of objects. Therefore, hidden thermal targets can be detected from the infrared image. Visible image (VIS) can obtain the spectral information of objects such as textural details and edges information. However, there are still many limitations to both IR and VIS images. For instance, VIS image is sensitive to environmental changes and IR image has low resolution. In order to obtain complementary information from IR and VIS image, IR and VIS image fusion technology is proposed [2].

Many IR and VIS image fusion methods have been proposed for many years. Overall, these methods could be classified into multi-scale transform-, sparse representation-, neural network-based methods, and hybrid models [3]. These methods have been developed for many years and widely applied to various fields. Therefore, we regard them as conventional fusion methods. Through studying complicated activity level measurement and fusion rules, these fusion approaches could enhance the fusion effect [4]. In multi-scale methods, it gets the different scale information of the source images by the multi-scale transformation. Then, the different scale information is fused according to the designed fusion rules. Finally, fused multi-scale information is inverse transformed to obtain the fused images. For example, Bavirisetti et al. proposed a multi-sensor image fusion based on fourth order partial differential equations (FPDE) [5]. Fourth order partial differential equations are applied to get approximation and detail information from source images. Principal component analysis was applied to obtain the optimal weights. Finally, FPDE fused the approximation and detail information by the optimal weights. Based on gradient transfer and total variation (TV) minimization, Ma et al. formulated the fusion problem as a TV minimization problem [6], where the data fidelity term keeps the main intensity distribution in the infrared image, and the regularization term preserves the gradient variation in the visible image. Zeng et al. proposed a fast fusion of visible and infrared images based on Fourier transform and difference minimization (FFVI) [7]. In sparse representation methods, Liu et al. proposed an adaptive sparse representation model for image denoising and fusion (ASR) [8]. It learned a set of more compact sub-dictionaries from a large number of high-quality image blocks. According to the gradient information of the target image block, sub-dictionaries was adaptively selected to fuse and denoise images. Neural networks based methods mainly used pulse coupled neural network (PCNN) or its variants to fuse images. At first, these methods decompose the source images into a series of image blocks and compute the feature maps of these image blocks. Then, PCNN is applied to fused these feature maps and construct the final fused image. Yin et al. proposed a new medical image fusion method in the PCNN domain [9]. They introduced a PCNN model into the fusion of high-frequency coefficients, and proposed a low-frequency fusion strategy that simultaneously addresses energy preservation and detail extraction. Nevertheless, manually design of activity level measurement and fusion rule is complicated. Therefore, the majority of conventional methods are suffered from relatively high computational costs, low robustness and implementation difficulty.

Deep learning technique has developed for many years and made many progresses in the field of computer vision. Through supervised learning or effective loss function, deep learning can generate a sophisticated nonlinear relationship between input and output. In the last three years, researchers have started to apply this technique to the field of image fusion. Deep learning-based methods can avoid the problems encountered by conventional fusion methods, because these methods can extract feature information of source images and merge these features into fused image through well-trained networks. The process of training networks is finished by software [10]. Compared with traditional methods, the fusion process of deep learning-based methods is simpler and more robust [11].

Generally, deep learning-based methods are divided into the following categories. (1) Methods united deep learning with conventional methods. These methods utilized the outstanding feature extraction ability of deep learning to obtain more complementary information. For instance, Liu et al. [12] transformed the source image into two parts. Convolutional sparse representation functions as a tool to merge these two parts. The final fusion result was generated by the inverse transformation. In [13], authors proposed a general image fusion model based on convolutional neural networks, termed as IFCNN. In this model, a simple encoder was utilized to obtain the feature information from source images. Fusion rule was designed to fuse these feature maps according to the category of source image. In the final step, the fused feature information was fed into decoder and the output of decoder was fused image. However, manual designing fusion rule still suffers from limitations of traditional methods. (2) Fusion methods with end-to-end learning structure. Typically, in the year of 2017, DeepFuse applied unsupervised deep learning architecture to multi-exposure image fusion [14]. DeepFuse designed a simple structure of CNN to get the feature maps of the multi-exposure image and reconstructed these feature maps into fused image. In DeepFuse, a measure of similarity between two images index (Structural Similarity Index, SSIM) was taken as the loss function which can force the fused image to contain more effective information from source images. In the year of 2018, Li et al. [15] applied densely connected convolutional networks (DenseNet) to IR and VIS image fusion, called as DenseFuse. In the process of training, the structure of DenseNet could obtain more complementary feature maps of the source image. DenseFuse regarded Visual Geometry Group’s network (VGGNet) [16] as a tool to obtain deep feature maps of source images. In 2020, Pan et al. applied a pre-trained dense block neural network to infrared and visual images [17]. Li et al. proposed a novel structure for image fusion [18]. Hou et al. presented a deep learning-based model for IR and VIS image fusion, termed as VIF-Net [19]. In their model, a hybrid loss function which was included SSIM and the total variation (TV) was designed. Recently, for the first time, the generative adversarial network (GAN) was used to fuse IR and VIS image (FusionGAN) [20]. For preserving more textural details from the VIS image in the fused image, the VIS image and the fusion result were fed into a discriminator. FusionGAN consisted of two parts: generator and discriminator. The generator was utilized to get a fused image that contains infrared thermal radiation information of the IR image. While the discriminator aimed at preserving texture information from the VIS image in the fused image. The adversarial relationship between generator and discriminator forced the fused image to maintain both textural details from the VIS image and thermal radiation information from the IR image. However, authors of FusionGAN just simply applied the structure of GAN to their model. FusionGAN cannot balance the weight of the generator and the discriminator in the training process, which may result in the problem of the loss of information from the source images. In addition to these representative deep learning image fusion algorithms, many of their variant algorithms have been proposed. In this paper, we will no longer introduce these methods [21,22,23,24].

**Figure 1:**
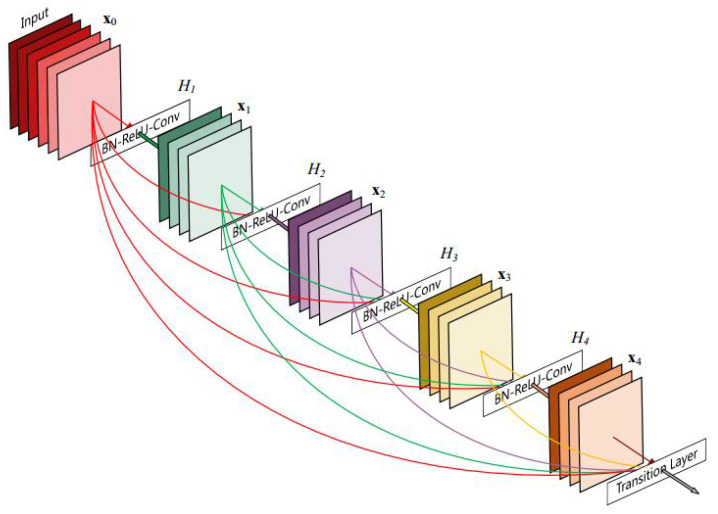
Structure of DenseNet [27].

For existing deep learning-based approaches, they aimed to design an appropriate loss function and novel architecture of the network. These two parts are essential in deep learning-based fusion methods which may result in better visual effect in the fused image [25,26]. As noted above, DeepFuse designed a simple structure of CNN and set structural similarity index as loss function to constrain their network. As we know, SSIM is a quality assessment which is widely used to assess the performances of fusion results. It calculates the correlation loss, contrast loss and brightness loss between the images. However, it is not enough for the networks to constrain their network by SSIM loss function that may lead to the loss of valid information, such as luminance, textural details and so on. It is pioneering in the field of image fusion that DeepFuse employed SSIM as the loss function. Consequently, following with DeepFuse, DenseFuse utilized SSIM to constrain their model. Especially, they used densely connected convolutional networks to extract the feature information, which is more efficient than convolutional neural network. However, for IR and VIS images, the feature information of these images varies greatly. Hence, the extraction process is difficult to perform in a uniform network. In FusionGAN, discriminator forced the generator to generate a fused image that includes more textural information from the VIS image. Whereas, they neglected the information from the infrared image. IFCNN designed fusion rules according to the type of the source image, but this way was still in the category of traditional methods. Overall, these works show the promising results. However, there are still some drawbacks. (a) The network architecture is an essential part in the process of feature extraction. Existing architecture is too simple to get valid features. Moreover, for multi-modality image fusion, using formed model to extract features of the source image with different characteristics will lead to the loss of textural details of source images. (b) Many existing approaches use neural networks to obtain feature information and reconstruct these fused feature maps. However, the fusion rule is still designed manually. Therefore, these methods still suffered from the limitations of conventional fusion methods. (c) For multi-modal image fusion, employing a single loss function cannot satisfy all kinds of source images. It is challenging to find an effective loss function. To tackle these defects, a novel unsupervised IR and VIS image fusion via DenseNet is proposed, named as TPFusion. In DenseNet, each dense block module uses information from all previous layers [27]. The structure of DenseNet is shown in Figure 1. By this architecture, DenseNet can reduce the parameters of networks, enhance the reusability of features from previous layers and alleviate the problem of gradient vanishing and model degradation. Moreover, motivated by DenseNet, we feed source images and texture information, which is obtained by Fourier transform into TPFusion. TPFusion can emphasize the texture information of the source image and prevent the loss of textural details in multi-modality image fusion. Consequently, the fused image will preserve more textural information. In the aspect of loss function, maxi-gradient loss and content loss are employed in our model, which enables the fused image to keep luminance and textural details of source images.

In general, the contributions of our work are listed as follows.

- We present a densely connected convolutional networks based unsupervised IR and VIS image fusion method. Moreover, based on the main idea of DenseNet, we extract texture information from the source image, which is set as auxiliary information to enable the fused result to contain more textural details.
- The loss functions we designed are according to the properties of IR and VIS image. Specifically, content loss and maxi-gradient loss can force the fusion result keeping the thermal radiation information from the IR image and the luminance information from VIS image, respectively.
- We establish a new registered IR and VIS image dataset with all conditions (including pedestrians, cars, military targets, buildings, and so on) which can advance the universality of deep learning-based image fusion methods.
- TPFusion is compared with existing representative image fusion methods. Qualitative and quantitative experiment results validate the effectiveness and universality of TPFusion.

The other parts of this research are arranged as follows. Details of TPFusion are illustrated in Section 2, including the structure of networks, loss functions and training details. In Section 3, we introduce the result of comparative experiments. Finally, the conclusion and future work is illustrated in Section 4.

## 2. Proposed Method

In this section, the details of TPFusion will be demonstrated, including the structure of the network, loss functions and training details.

### 2.1. The Structure of TPFusion

**Figure 2:**
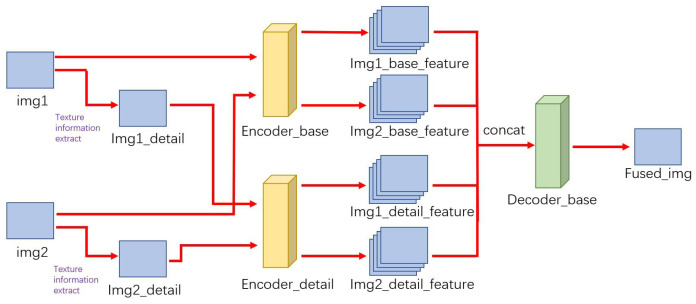
The overall architecture of the proposed TPFusion.

The overall structure of TPFusion is shown in Figure 2. The proposed model consists of two encoders and a decoder. The pre-registered IR and VIS images are named as img1 and img2, respectively. We get textural detail information of the source image by Fourier transform, which is a general way to get the high frequency component. The high frequency component of image denotes the place of the image where the intensity (brightness/grayscale) changes dramatically. To be specific, the textural detail information is obtained as following:

(1) $$F\left(u,v\right)=\sum_{x=0}^{M-1}\sum_{y=0}^{y=0}f\left(u,v\right)exp\left[-j2\pi \left(\frac{ux}{M}+\frac{vy}{N}\right)\right]$$

(2) $$F_{high}\left(u,v\right)=\{\;\begin{array}{c} f\left(x,y\right),\;\mathrm{if}\;0<x<\frac{u}{2}\cup 0<y<\frac{v}{2}\\ 0,\;\mathrm{else} \end{array}$$

(3) $$f_{high}\left(x,y\right)=\frac{1}{MN}\sum_{x=0}^{M-1}\sum_{y=0}^{N-1}F_{high}\left(u,v\right)exp\left[j2\pi \left(\frac{ux}{M}+\frac{vy}{N}\right)\right]$$

We utilize Fourier transform to get frequency domain information from the image, and filter out low frequency information. The high frequency component of the image is obtained by inverse Fourier transform. Then, Encoder-base is used for extracting feature layers of the source image. In the meantime, the detail feature layers are obtained from textural detail information by Encoder-detail. Due to the fact that characteristics of source images and textural details information are different, for extracting more effective information, we use the different feature extraction encoder (Encoder-base and Encoder-detail) to get features from the source image and textural details of source images. Although Encoder-base and Encoder-detail have the same structure. However, through the training step, the encoders have different parameters of network that can obtain diverse feature information from source images. We concat these feature maps and feed them into the decoder. Finally, the output of the decoder is the fused image.

**Figure 3:**
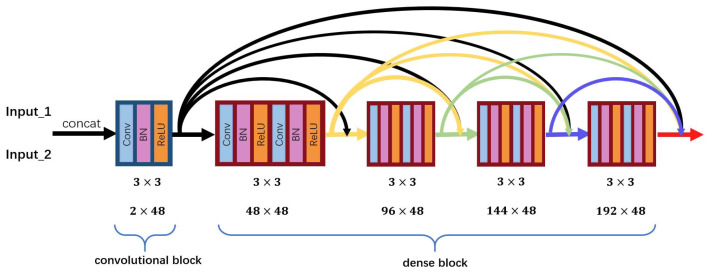
The structure of Encoder-detail and Encoder-base.

The Encoder-base and Encoder-detail have the same structure, which is illustrated in Figure 3. Encoder has two parts, including common convolutional block and dense block. The input of Encoder-base and Encoder-detail is concatenated img1 and img2, img1-detail and img2-detail, respectively.

**Figure 4:**
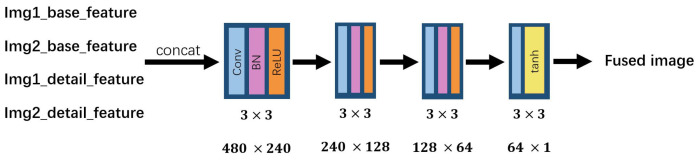
The structure of the decoder.

The convolutional block is utilized to extract low-level features. For suiting input with any size, we set all the size and stride of convolutional kernel as 3 × 3 and 1, respectively. For tackling the problem of gradient exploding and improving the stability during training process, batch normalization and ReLU are applied to TPFusion. In Figure 3, 2 × 48 means the channel of input of convolutional block is 2 and the output is 48. In dense block, we apply a densely connected network [27] to obtain the deep features of input. Two convolutional layers in each densely connected layer were employed that enable dense blocks to obtain higher level features, and these features are used in short direct connection. Through this way, we can reduce the number of parameters in dense block and accelerate the training step. Densely connected network addresses the problem of vanishing gradients and strengthens feature propagation. Hence, comparing with a convolutional neural network, it can preserve more depth features. We concat the deep features from Encoder-base and Encoder-detail and fed these features into the decoder. As shown in Figure 4, the decoder consists of four convolutional layers that are used to fuse these features and generate the fused image. The settings of parameters in the decoder are the same with the encoder.

### 2.2. Loss Function

As we know, deep learning utilizes a loss function or ground truth to constrain their models in the training step. In the field of IR and VIS image fusion, it is tough to find labeled fused images (ground truth). Therefore, the loss function is vital for IR and VIS image fusion. Our TPFusion aims at reserving the textural details and thermal radiation information from source images. Consequently, the loss function of TPFusion consists of content loss ($L_{con}$) and the maxi-gradient loss ($L_{maxi-grad}$).

(4) $$LOSS=L_{con}+\gamma L_{maxi-grad},$$

where γ is used to control the trade-off between the maxi-gradient loss and pixel intensities loss. $L_{con}$ is defined as follows,

(5) $$L_{con}=\frac{1}{h\cdot w}\left[sum{\left|I_{f}-I_{vis}\right|}_{F}+sum{\left|I_{f}-I_{ir}\right|}_{F}\right].$$

$I_{ir}$, $I_{vis}$ and $I_{f}$ presents the fused image, visible image and infrared image, respectively. *h* and *w* denotes the height and width of the source image. sum(∗) is element summation of the input. ${\left|*\right|}_{F}$ conveys the matrix Frobenius norm, and $L_{maxi-grad}$ is calculated as:

(6) $$L_{maxi-grad}=\frac{1}{h\cdot w}sum\left({\left|\vec{I_{f}}-maxi\left(\vec{I_{vis}},\vec{I_{ir}}\right)\right|}_{l1}\right),$$

where the $\vec{*}$ is the gradient operation. In order to preserve the textural information of visible image and infrared image in the fused image, we calculate the gradient of the source images, and use maxi(∗) to obtain the maxi gradient of source image. ${\left|*\right|}_{l1}$ denotes the $l_{1}$ distance.

### 2.3. Training

**Figure 5:**
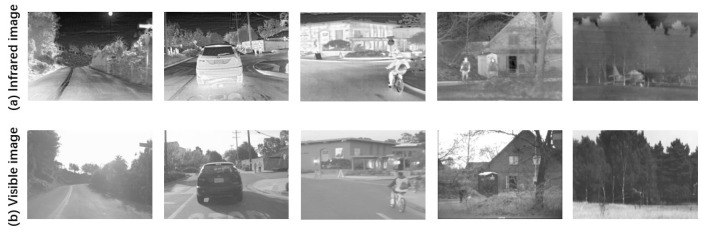
Infrared and visible images training data sets.

As for training dataset, our team has disclosed a new VIS-IR image dataset RoadScene (including 221 image pairs). There are many similar scene images in the RoadScene. Therefore, for improving the efficiency of network model training, we select 88 image pairs with a variety of scenes, including pedestrians in natural scenes, road scenes, cars, military targets, buildings, etc. 44 image pairs set as training dataset and the other images are regarded as testing dataset. Five pairs of infrared and visible images in the training dataset are presented in Figure 5.

For expanding the amount of source data, maximum filter is used to down-sample the source images and crop these data into 7936 patch pairs with the size of 84×84. RMSPropOptimizer is applied to update the parameters of TPFusion. After parameter ablation experiments, the network parameters are finally set as follows, $learning-rate=10^{-4}$; EPOCHES=6; BATCH−SIZE=12.

## 3. Experiment Results and Analysis

We have conducted qualitative and quantitative experiments against seven image fusion methods. Three of them are deep learning-based methods, and the rest are traditional approaches. All the experiments are conducted in our own server, and the hardware information of our server are as follows: 3.6 GHz Intel Core CPU I9-9900K, GTX 2080Ti, and 64 GB memory.

### 3.1. Comparison Methods

We select seven representative IR and VIS image fusion methods to compare with TPFusion, including FPDE [5], GTF [6], FFVI [7], ASR [8], DenseNetFuse [17], DenseFuse [15] and FusionGAN [20].

### 3.2. Qualitative Comparisons

**Figure 6:**
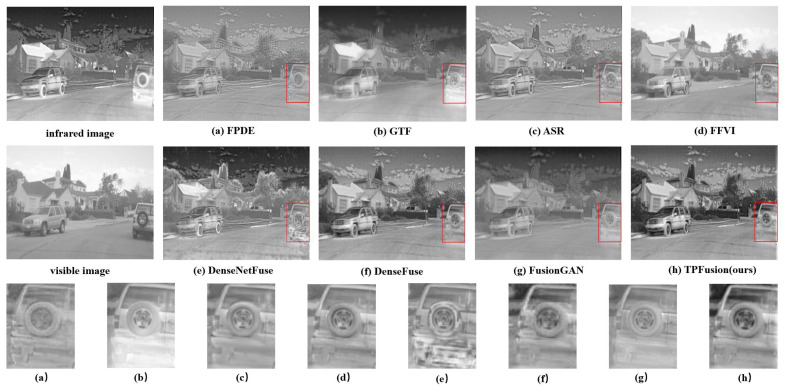
Qualitative comparison results on image sequence 1.

**Figure 7:**
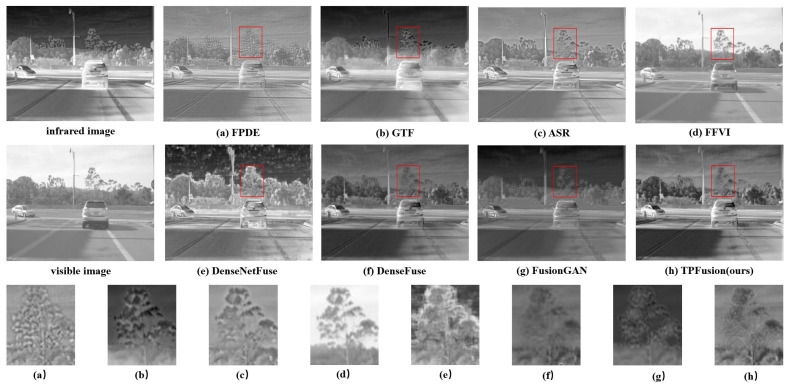
Qualitative comparison results on image sequence 2.

**Figure 8:**
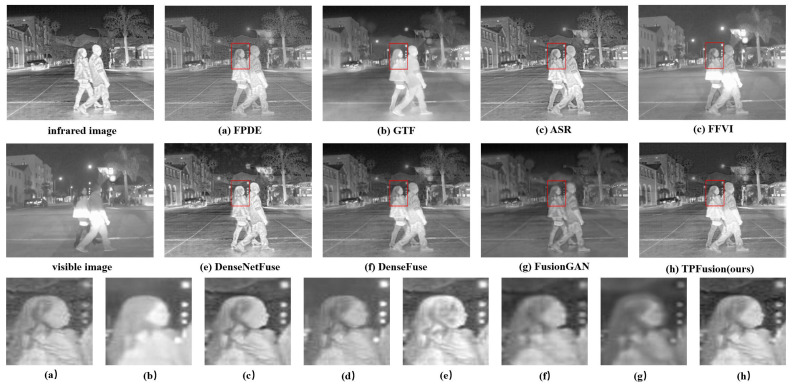
Qualitative comparison results on image sequence 3.

In this section, we perform a qualitative comparison experiment and select three fused image sequences with an obvious contrast effect from the comparison results. Qualitative comparison results are illustrated in Figure 6, Figure 7 and Figure 8. In the qualitative comparison experiment, we assess the fusion result from two sides, the textural details and the general image visual effect. In the view of general visual effects, the results of FPDE and ASR show high brightness and low contrast ratio. The fused images of GTF, DenseNetFuse and FusionGAN lose some texture details. FFVI pays more attention to the visible image, which may lead to the loss of thermal radiation information. For example, in Figure 6d, the cloud of infrared images is missing. DenseFuse and our method both generate well-proportioned light distribution and high contrast ratio. However, the fused image obtained by DenseFuse suffers from the local blur problem. Consequently, from the general visual effect, TPFusion is more consistent with human visual perception. As for the aspect of the textural details, especially in the red box that we marked in the fused image, the comparison methods have all lost some textural details. Specifically, Figure 6 shows that the textural details of the wheel in TPFusion own the clearest visual effect with high contrast. In the red box of Figure 7, our work displays that the texture of the tree is clearer. As for the face we marked in Figure 8, facial features of our result exhibit clearer details.

### 3.3. Quantitative Comparisons

In quantitative experiments, we select six commonly used quantitative metrics, including SSIM [28], EN [29], MG [30], CC [31], EI and SCD [32], to assess the effect of comparison approaches. These six metrics are introduced below.

*Structural similarity index measure (SSIM).* In 2004, Wang et al. presented a universal fusion quality metric named SSIM [28]. There are three components in SSIM, including the loss of contrast distortion, luminance and correlation. SSIM is mathematically calculated as follows:

(7) $$SSIM_{S,F}=\sum_{s,f}\frac{2\mu_{s}\mu_{f}+c_{1}}{\mu_{s}^{2}+\mu_{f}^{2}+c_{1}}\cdot \frac{2\sigma_{s}\sigma_{f}+c_{2}}{\sigma_{s}^{2}+\sigma_{f}^{2}+c_{2}}\cdot \frac{\sigma_{sf}+c_{3}}{\sigma_{s}\sigma_{f}+c_{3}}.$$

$SSIM_{S,F}$ calculates the metric between source image *S* and fused image *F*; *s* and *f* means the image blocks of *S* and *F* in a sliding window; $\sigma_{xf}$ indicates the covariance of *x* and *f*; $\sigma_{*}$ denotes the standard deviation (*SD*); and $\mu_{*}$ presents the mean operation of ∗. C1, C2, and C3 are the parameters for stability; when C1=C2=C3=0, the SSIM can be regarded as the universal image quality index. A large SSIM indicates the better performance in structural similarity.

*Entropy (EN). EN* is a measurement of information theory, which is mainly used to assess the amount of information of images [29]. The EN is defined as the following:

(8) $$EN=-\sum_{x=0}^{255}p_{x}log_{2}\left(p_{x}\right),$$

where *x* means the gray level and $p_{x}$ is the normalized histogram with the gray level *x* of the image. A larger EN means the richer information the image has.

*Mean gradient (MG).* The metric of *MG* presents the gradient information of images [30]. MG is calculated as follows:

(9) $$MG=\frac{1}{\left(W-1)(H-1\right)}\sum_{x=1}^{W-1}\sum_{y=1}^{H-1}\sqrt{\frac{{\left(F\left(x,y\right)-F\left(x-1,y\right)\right)}^{2}+{\left(F\left(x,y\right)-F\left(x,y-1\right)\right)}^{2}}{2}},$$

where the *H* and *W* means the size of the image (height and width). F(x,y) indicates the grey level of (x,y). Gradient information represents the textural details of the image. Therefore, the larger value of MG indicates the more textural details in the image.

*Correlation coefficient (CC). CC* calculates the Pearson correlation coefficient of source images and fused images, which can be defined as follows [31]:

(10) $$CC=\frac{\sum_{x=1}^{M}\sum_{y=1}^{N}\left(X\left(x,y\right)-\bar{X}\right)\left(F\left(x,y\right)-\bar{F}\right)}{\sqrt{\left(\sum_{x=1}^{M}\sum_{y=1}^{N}{\left(X\left(x,y\right)-\bar{X}\right)}^{2}\right)\left(\sum_{x=1}^{M}\sum_{y=1}^{N}{\left(F\left(x,y\right)-\bar{F}\right)}^{2}\right)}}.$$

$\bar{*}$ denotes the mean value operation. A larger CC indicates the more similar of two images, which means the fused image has more information from source image.

*Edge intensity (EI). EI* denotes the edge information of image. *EI* can be calculated as follows:

(11) $$EI=\frac{\sum_{x=1}^{M}\sum_{y=1}^{N}\sqrt{S_{x}{\left(x,y\right)}^{2}+S_{y}{\left(x,y\right)}^{2}}}{M\cdot N},$$

where the $S_{x}$ is the Sobel matrix. The larger the value of EI indicates the higher contrast of the image and the richer edge detail.

*The sum of correlations of differences (SCD).* In the theory of SCD, the contribution of source images S1 in the fused image *F* is approximate to the difference between the other source image S2 and the fused image *F* [32]. The differences (D1 and D2) are calculated as the following:

(12) $$D_{1}=F-S_{2},$$

(13) $$D_{2}=F-S_{1}.$$

SCD is defined as:

(14) $$SCD=r\left(D_{1},S_{1}\right)+r\left(D_{2},S_{2}\right),$$

where the $r(D_{k},S_{k})$ is to calculate the similarity between $D_{k}$ and $S_{k}$, which is defined as the following:

(15) $$r\left(D_{k},S_{k}\right)=\frac{\sum_{i}\sum_{j}\left(D_{k}\left(i,j\right)-\bar{D_{k}}\right)\left(S_{k}\left(i,j\right)-\bar{S_{k}}\right)}{\sqrt{\left(\sum_{i}\sum_{j}{\left(D_{k}\left(i,j\right)-\bar{D_{k}}\right)}^{2}\right)\left(\sum_{i}\sum_{j}{\left(S_{k}\left(i,j\right)-\bar{S_{k}}\right)}^{2}\right)}}.$$

A larger value of SCD indicates the higher correlations of source images and fused image, which means that the fused image preserves more complementary information of source images.

**Figure 9:**
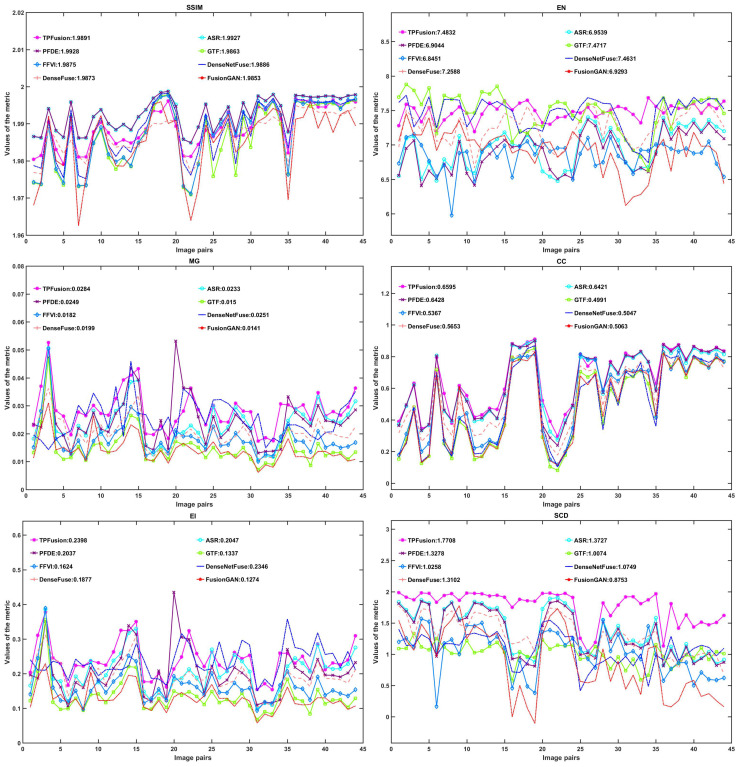
Quantitative comparisons of the six metrics.

We regard the above mentioned metrics as index of quantitative experiments. The fused images of comparison experiments, the assessment results are shown in Figure 9, and the mean value of each metrics are presented in Table 1. As shown in Table 1, we mark the largest value and the second place of value with the color of red and blue, respectively. Table 1 indicates that TPFusion exhibit an ideal fusion result. TPFusion owns the largest mean value in SCD, EN, MG, EI and CC. TPFusion gets the third place in SSIM, and the SSIM of ASR and FPDE has the first and second place, respectively. As mentioned above, SSIM can measure images from three aspects, including contrast, luminance and correlation. Nevertheless, observing Figure 6, Figure 7 and Figure 8, we find that ASR and FPDE have the phenomenon of relatively high luminance, which will lead to a high value of SSIM. However, relatively high luminance of ASR and FPDE is abnormal and may result in the loss of image information. Apart from these two methods, our method achieves the first place of the other methods.

### 3.4. Ablation Experiment

For proving the effectiveness of contributions in our framework, ablation experiments have been performed. To be specific, in ablation experiment 1, the fusion results of TPFusion without Encoder-detail is presented. In the ablation experiment 2, we removed the short direct connection of original densely connection network to get conventional convolutional neural network which was used to extract deep features of the source image. In addition, we implement a parameter study on key weight in TPFusion. To be specific, we employ the weight γ to balance the maxi-gradient loss and content loss. In the subsequence ablation experiments, we present the fusion results with different value of γ. In TPFusion, γ is 1. We set the weight γ as 5 and 10 in the ablation experiment 3 and 4, respectively.

**Figure 10:**
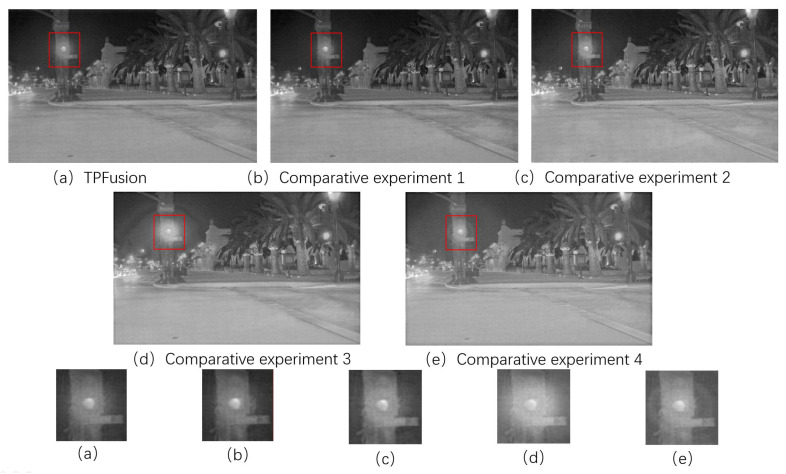
The qualitative results of the ablation experiments.

The qualitative results of ablation experiment are presented in Figure 10. From the aspect of general image visual effect, Figure 10a–c have a higher contrast ratio. The overall brightness of Figure 10d,e is too high. As for detail information, in the red marked box, the result of Figure 10a is the clearest and the light halo is closer to reality.

Quantitative result is presented in Table 2 and Table 3. As illustrated from Table 2, except for CC, our work TPFusion acquires the best values among the comparative experiment 1 and 2. It indicates the effectiveness of our method. From Table 3, our work gets the first place of five metrics, including EN, MG, EI and SCD. However, from the qualitative results we can find that ablation experiment 3 and 4 have the phenomenon of high illumination which will result in a high value of SSIM. Consequently, in general, it is the best choice to set γ as 1.

## 4. Conclusions and Future Work

We present a novel network architecture for IR and VIS image fusion in this article, called TPFusion. We extract textural information of source images, which is regarded as auxiliary information. Together with source images, these textural details are fed into a densely connected network to generate a fused image. Through the help of additional auxiliary information, it can avoid the vanishing of textural details during the training process of deep networks. Consequently, the fused image can obtain more textural information of source images. Moreover, we design an effective loss function to constrain the whole network. From the aspect of qualitative and quantitative analysis, our method acquires promising performance among the other five representative IR and VIS image fusion methods. In the future research, we aim at designing the variants of a densely connected network or other neural networks to fuse multi-modal images. Moreover, the mean value of processing time of this work is about 0.1 s. Our further research is aim at applying fusion methods to the practical engineering.

## Figures and Tables

**Table 1:** Mean value of quantitative comparisons of the six metrics.

Method	SSIM	EN	MG	CC	EI	SCD
TPFusion	1.9891	7.4832	0.0284	0.6492	0.2398	1.7708
ASR	1.9927	6.9539	0.0233	0.6421	0.2047	1.3727
FPDE	1.9928	6.9044	0.0249	0.6428	0.2037	1.3278
GTF	1.9863	7.4717	0.0150	0.4991	0.1337	1.0074
FFVI	1.9875	6.8451	0.0182	0.5367	0.1624	1.0258
DenseNetFuse	1.9886	7.4631	0.0251	0.5047	0.2346	1.0749
DenseFuse	1.9873	7.2588	0.0199	0.5653	0.1877	1.3102
FusionGAN	1.9853	6.9293	0.0141	0.5063	0.1274	0.8753

**Table 2:** Mean value of quantitative comparisons of ablation experiment.

Method	SSIM	EN	MG	CC	EI	SCD
TPFusion	1.9891	7.4832	0.0284	0.6492	0.2398	1.7708
ablation experiment 1	1.9888	7.3417	0.0245	0.6592	0.2027	1.7435
ablation experiment 2	1.9887	7.3053	0.0244	0.6583	0.2026	1.7061

**Table 3:** Mean value of quantitative comparisons of parameter study.

Method	SSIM	EN	MG	CC	EI	SCD
TPFusion	1.9891	7.4832	0.0284	0.6492	0.2398	1.7708
ablation experiment 3	1.9911	7.2668	0.0241	0.6569	0.2016	1.6557
ablation experiment 4	1.9916	7.2343	0.0227	0.6532	0.1901	1.6083

## Data Availability

Not applicable.
